# First reported case of safe and efficacious use of tocilizumab for treatment of hyperinflammatory syndrome associated with COVID‐19 in an allogeneic stem cell transplant patient

**DOI:** 10.1002/jha2.132

**Published:** 2020-12-08

**Authors:** Nicholas Fordham, Emma Baker, Daniel Forton, Matthias Klammer, Kamal Patel, Dara Qadir, Yasmin Reyal, Fenella Willis, Mickey B.C. Koh

**Affiliations:** ^1^ Haematology Department St George's University Hospitals NHS Foundation Trust London UK; ^2^ St Catherine's College University of Oxford Oxford UK; ^3^ Institute of Infection and Immunity St George's University of London UK; ^4^ Department of Gastroenterology and Hepatology St George's University Hospitals NHS Foundation Trust London UK; ^5^ Cell Therapy Programme Health Sciences Authority Singapore

**Abbreviations**
CMV: Cytomegalovirus
CRP: C‐Reactive Protein
EBV: Epstein‐Barr Virus
GvHD: Graft‐versus‐host disease
HSCT: Haematopoietic stem cell transplant
LDH: Lactate dehydrogenase

## 1 CASE DESCRIPTION

We present the first published case demonstrating the safe and efficacious use of tocilizumab in a haematopoietic stem cell transplant (HSCT) patient with COVID‐19. The 34‐year‐old male with a diagnosis of Hodgkin lymphoma had been treated with three lines of chemo‐immunotherapy, however due to relapse and subsequent refractory disease, he underwent an HSCT with a matched unrelated donor. He was discharged from hospital 4.5 months prior, with Grade 2 skin graft‐versus‐host disease (GvHD), treated with topical steroids and cyclosporine.

**FIGURE 1:**
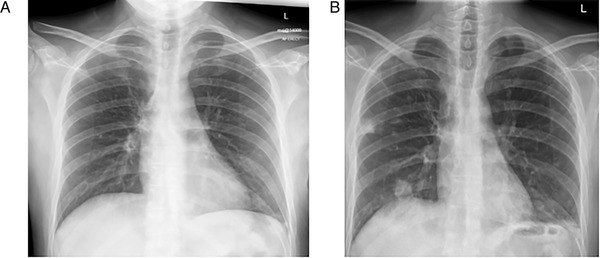
The progression of chest X‐ray changes. (A) Chest radiograph on admission and (B) with increasing oxygen requirement

He presented to the emergency department with a history of diarrhoea, cough, and fever (>38°C), and was commenced on broad‐spectrum antibiotics. His chest X‐ray was normal (Figure 1A), and swabs for SARS‐CoV2 RNA were negative three times, as were swabs for alternative respiratory viruses. Throughout his admission, repeated blood cultures, stool pathogen screens, and atypical pneumonia screens were negative. Cytomegalovirus (CMV) and adenovirus PCRs were negative, and an Epstein‐Barr virus (EBV) PCR revealed a low level reactivation of 230 IU/mL. His cyclosporine was weaned and discontinued by day nine of admission.

**FIGURE 2:**
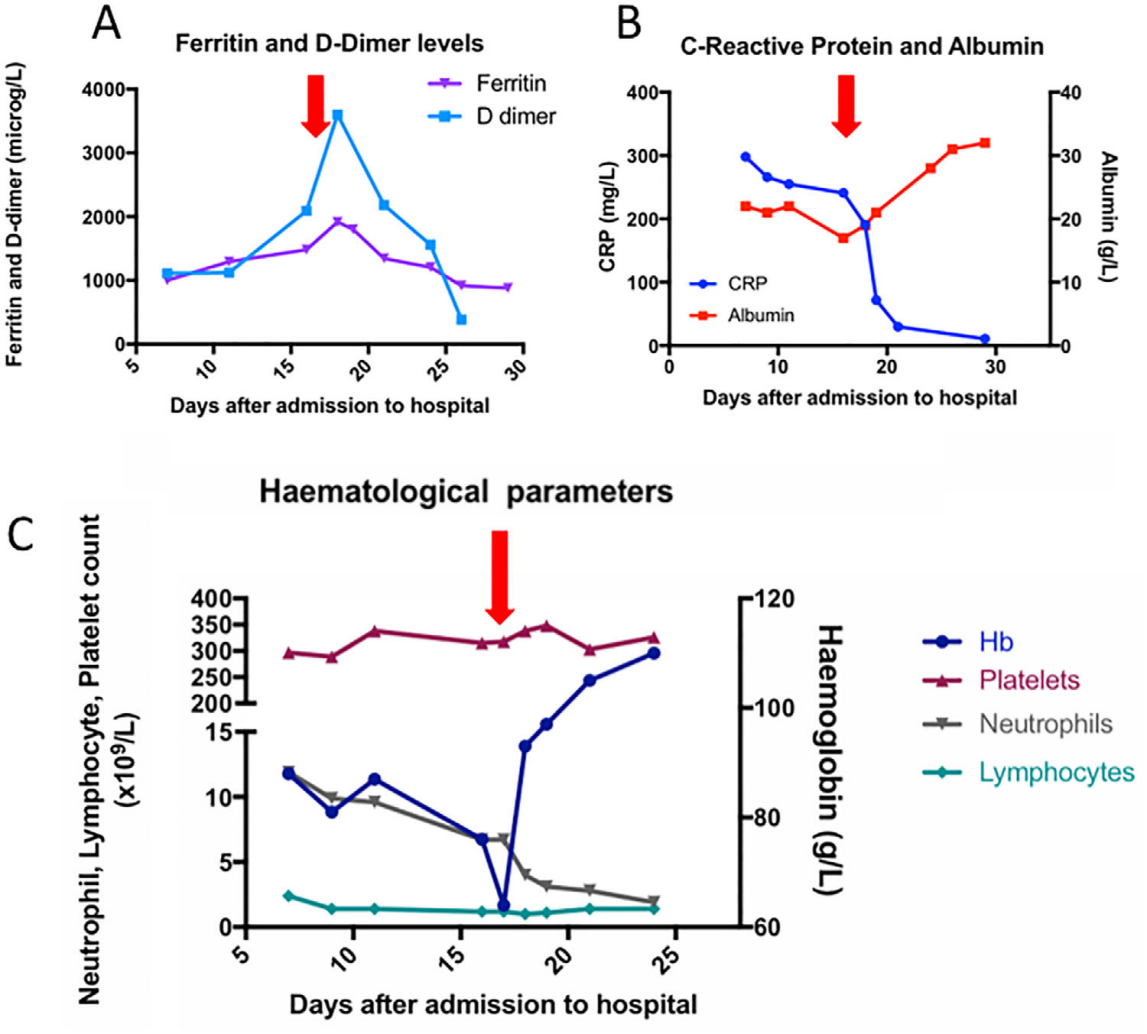
Laboratory markers. (A and B) Inflammatory markers and (C) full blood count parameters. Red arrows mark tocilizumab treatment

On day 6, he suffered ongoing fevers despite antibiotics, and a repeat chest X‐ray suggested COVID‐19 (Figure 1B). A fourth COVID‐19 swab returned positive. His inflammatory markers worsened (Figure 2A and B), with rising ferritin. He developed anaemia, requiring a single top‐up transfusion, and lymphopenia (Figure 2C), and by day 16 he had developed an increasing oxygen requirement up to 10 L/min.

Computerised tomography (CT) scanning confirmed extensive ground glass changes compatible with COVID‐19 and no alternative pathology. In view of his increasing oxygen requirement, persistent fevers and hyperinflammatory state – rising ferritin, elevated lactate dehydrogenase (LDH), C‐reactive protein (CRP) and D‐dimers – the patient was given 8 mg/kg of tocilizumab (Actemra; Roche, UK) as part of a hospital‐approved compassionate use programme.

Following tocilizumab, his fevers abated within 24 hours. There was rapid improvement in haematological and inflammatory parameters (Figure 2A–C). The patient noticed a significant clinical improvement; however, his oxygen requirement demonstrated a more gradual course and over 2 weeks was weaned back to 4 L/min. Antibiotics were discontinued after 7 days. His chest X‐ray remained compatible with COVID‐19, but did not worsen and, having discontinued his cyclosporine, there was no flare up of his skin GvHD. His haemoglobin showed a marked improvement, despite no further transfusions, and his albumin normalised. There was no reactivation of CMV, EBV or adenovirus, following tocilizumab.

Although he progressively improved, he required oxygen therapy for around 6 weeks. His COVID‐19 swab remains positive 6 weeks later, and his chest X‐ray continues to show changes consistent with COVID‐19.

## 2 DISCUSSION

COVID‐19 is a novel coronavirus, currently causing a global pandemic. Although initial reports from China focussed on the respiratory complications [1, 2], subsequent experience has demonstrated that patients’ clinical course is heterogeneous with a proportion of patients developing a hyperinflammatory syndrome marked by persistent fevers and cytokine storm [3, 4]. A retrospective analysis of patients from China suggested that raised markers of inflammation, particularly CRP and IL‐6, were poor prognostic markers in COVID‐19 [2, 5], and another study demonstrated the prognostic significance of elevated D‐dimers [6].

Although immunosuppression with corticosteroids was initially thought counterproductive [5], in the presence of hyperinflammation, targeted immunomodulation may improve mortality [7]. IL‐6 is an important regulator of the human inflammatory response, including induction of CRP, and may therefore be a useful target [8]. Previous reports suggested that tocilizumab may have benefit in COVID‐19 [3, 9], with reduction of biochemical parameters, although some patients required multiple doses to show clinical improvement [9].

There is concern, however, in immunosuppressed individuals that further suppression of the immune response may predispose to sepsis or unchecked viral replication. In addition, such treatments may impair the identification of sepsis due to a lack of clinical and biochemical markers. Targeting of individual cytokines may have some benefit, despite the above concerns [10]. The IL‐6 inhibitor tocliziumab has been used successfully in cytokine release syndrome following chimeric antigen receptor T‐cell therapy (CAR‐T) [11], and IL‐1 blockade with anakinra has proven beneficial in patients with a hyperinflammatory state secondary to sepsis [12].

Case reports have demonstrated the safety of tocilizumab for COVID‐19 in patients with haematological malignancies and solid organ transplant recipients [13, 14], however this has not yet been reported in HSCT patients. Patients post‐HSCT demonstrate marked immune suppression due to ongoing therapy, presence of GvHD as well as delayed immune reconstitution. Our patient had a recent transplant, was taking cyclosporine on admission and had antecedent GvHD. Nonetheless, this patient was considered for tocilizumab use due to his deteriorating clinical condition associated with clear signs of hyperinflammation.

In this protocol, patients meeting the clinical criteria for access to tocilizumab are selected based primarily on a hyperinflammatory presentation needing at least three of the following: elevated D‐dimer, rising CRP, ferritin > 1000 ng/mL, elevated LDH, in addition to confirmed SARS‐CoV‐2 with radiographic evidence of pneumonia and increasing oxygen requirement. There should be an absence of bacterial infection and each case is assessed by an independent multidisciplinary panel set up specifically for this, and adhered to WHO guidelines [15]. Our patient showed elevation of all four inflammatory markers and fulfilled the criteria for treatment. Following the treatment, these markers were monitored, and he showed a rapid improvement after a single dose of tocilizumab.

The other point of note is the continued persistence of the COVID‐19 virus in our patient with swab positivity for SARS‐CoV2 RNA. This is not unusual considering similar experiences with other respiratory viruses where HSCT patients can continue to remain positive on testing up to 3 months since infection. This correlates with our patient's protracted clinical course, with slow recovery and weaning off oxygen. Significantly, there was no further viral reactivation or bacterial infection following tocilizumab treatment.

In conclusion, we illustrate the potential safety and efficacy of tocilizumab in HSCT patients. Coupled with clinical and biochemical improvement, we suggest that further research on tocilizumab in patients with haematological malignancies and HSCT recipients with COVID‐19 is warranted, and these patients should not be excluded from clinical trials for immune modulation.

## AUTHOR CONTRIBUTIONS

Nicholas Fordham and Mickey B.C. Koh conceived and edited the manuscript. Emma Baker, Daniel Forton, Kamal Patel, Matthias Klammer, Dara Qadir, Yasmin Reyal and Fenella Willis were involved in the case and drafting the manuscript. All authors approved the final manuscript.

## PATIENT CONSENT

A written consent was obtained from the patient.

## CONFLICT OF INTEREST

The authors declare that there is no conflict of interest.

## Data Availability

The data that support the findings of this study are available on request from the corresponding author. The data are not publicly available due to privacy or ethical restrictions.
